# Recent advances in prostate cancer research: large-scale genomic analyses reveal novel driver mutations and DNA repair defects

**DOI:** 10.12688/f1000research.14499.1

**Published:** 2018-08-02

**Authors:** Sander Frank, Peter Nelson, Valeri Vasioukhin

**Affiliations:** 1 Division of Human Biology, Fred Hutchinson Cancer Research Center, Seattle, WA 98109, USA; 2 Division of Clinical Research, Fred Hutchinson Cancer Research Center, Seattle, WA 98109, USA; 3 Departments of Medicine and Urology, University of Washington, Seattle, WA 98195, USA; 4 Department of Pathology, University of Washington, Seattle, WA 98195, USA

**Keywords:** prostate cancer, sequencing, xenograft, immunotherapy, 3D culture, PARP, BRCA

## Abstract

Prostate cancer (PCa) is a disease of mutated and misregulated genes. However, primary prostate tumors have relatively few mutations, and only three genes ( *ERG*, *PTEN*, and *SPOP*) are recurrently mutated in more than 10% of primary tumors. On the other hand, metastatic castration-resistant tumors have more mutations, but, with the exception of the androgen receptor gene ( *AR*), no single gene is altered in more than half of tumors. Structural genomic rearrangements are common, including *ERG *fusions, copy gains involving the *MYC* locus, and copy losses containing *PTEN*. Overall, instead of being associated with a single dominant driver event, prostate tumors display various combinations of modifications in oncogenes and tumor suppressors. This review takes a broad look at the recent advances in PCa research, including understanding the genetic alterations that drive the disease and how specific mutations can sensitize tumors to potential therapies. We begin with an overview of the genomic landscape of primary and metastatic PCa, enabled by recent large-scale sequencing efforts. Advances in three-dimensional cell culture techniques and mouse models for PCa are also discussed, and particular emphasis is placed on the benefits of patient-derived xenograft models. We also review research into understanding how ETS fusions (in particular, *TMPRSS2-ERG*) and *SPOP* mutations contribute to tumor initiation. Next, we examine the recent findings on the prevalence of germline DNA repair mutations in about 12% of patients with metastatic disease and their potential benefit from the use of poly(ADP-ribose) polymerase (PARP) inhibitors and immune modulation. Lastly, we discuss the recent increased prevalence of AR-negative tumors (neuroendocrine and double-negative) and the current state of immunotherapy in PCa. AR remains the primary clinical target for PCa therapies; however, it does not act alone, and better understanding of supporting mutations may help guide the development of novel therapeutic strategies.

## Introduction

Prostate cancer (PCa) is the most commonly diagnosed non-skin cancer in American men and is estimated to account for about 30,000 deaths this year in the USA and at least 10 times as many worldwide ^1, 2^. The disease is curable when locally confined, but treatment options are limited for metastatic disease. First recognized in the 1940s as an effective therapy for metastatic PCa ^3^, androgen deprivation remains the primary option for patients with advanced disease; however, tumors invariably relapse into incurable metastatic castration-resistant PCa (mCRPC) ^4^. Further targeting of the androgen receptor (AR) axis with more effective drugs has extended survival by a few months but leads to resistance, including an increase in once-rare neuroendocrine and non-neuroendocrine/AR-negative tumors ^5^. Owing to recent large-scale sequencing efforts, there is now a better understanding of the genomic landscape of PCa, including characterization of lower-frequency but nonetheless important mutations in *SPOP* and DNA repair genes (for example, *BRCA2*).

This review covers some of the recent advances in understanding PCa, including identification and targeting of key genetic aberrations ( *ERG*, *SPOP*, and DNA repair defects), improvements in disease models, the emergence of AR-negative disease, and current immunotherapy strategies. Although AR signaling remains the ultimate driver of most PCa, tumors show an assortment of additional alterations that help promote disease progression and at the same time provide new opportunities for targeting this resilient disease. Although we sought to cover a wide range of topics, many fell beyond the scope of this report. However, several of those important themes can be found in previous reviews, including epigentics ^6, 7^, diet ^8^, tumor metabolism ^9, 10^, biomarkers ^11^, microRNAs ^12, 13^, the role of the microenvironment ^14, 15^, and racial disparities ^16^.

## Genomic analysis of primary tumors

**Figure 1.**
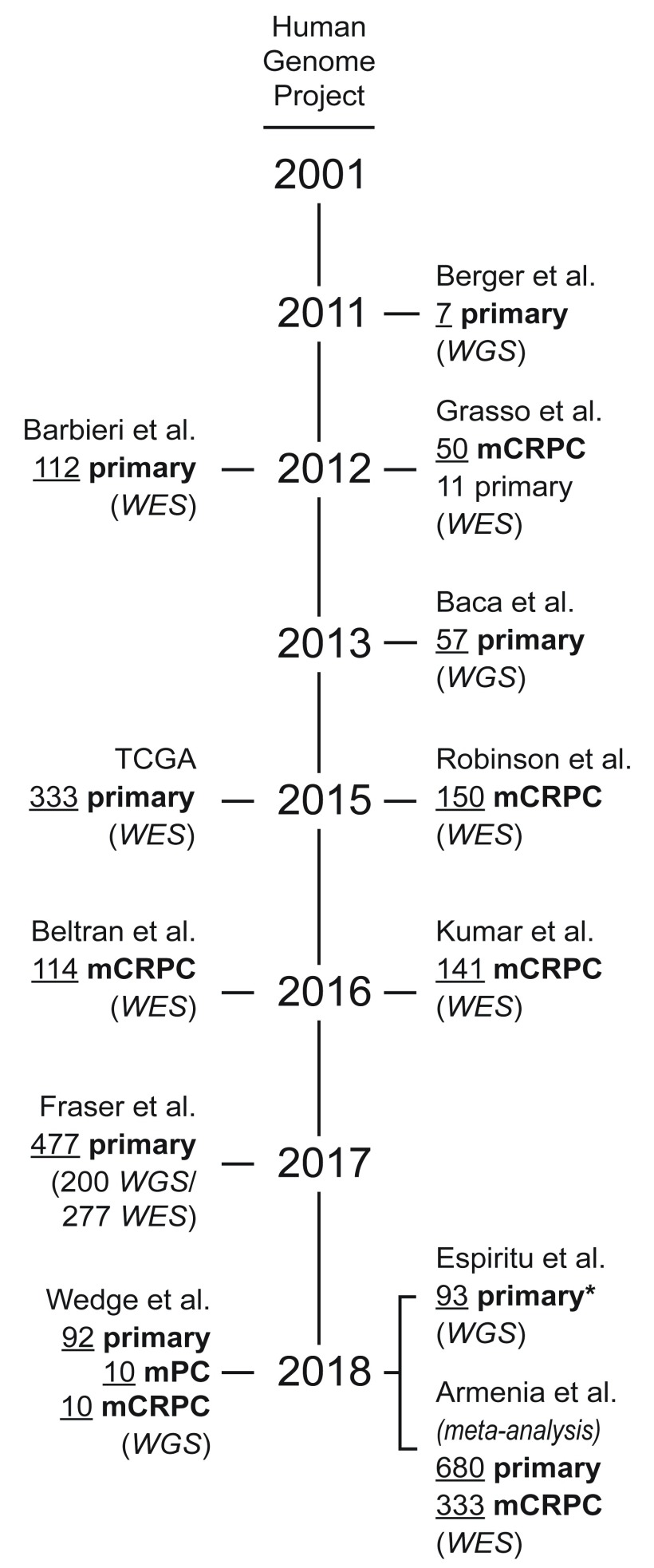
Timeline (not to scale) of key prostate cancer whole genome sequencing and whole exome sequencing studies ^19– 26, 27– 30^. One of these studies included 10 hormone treatment-naïve metastatic tumors (metastatic prostate cancer) ^24^. *A total of 293 primary tumors were analyzed in this study, but 200 were included in a previous study and not included here ^23^. The study by Armenia *et al*. ^26^ is a uniform re-analysis of previous whole exome sequencing studies, including many of those listed here. mCRPC, metastatic castration-resistant prostate cancer; mPC, metastatic prostate cancer; TCGA, The Cancer Genome Atlas; WES, whole exome sequencing; WGS, whole genome sequencing.

Analysis of PCa at the genome level began around the turn of the century with a wide range of studies using a combination of techniques, such as comparative genomic hybridization, DNA microarray, and targeted sequencing ^17, 18^. Whole genome sequencing (WGS) efforts began around 2011, including a project that performed WGS of seven primary tumors ( Figure 1) ^19^. Over the next 2 years, whole exome sequencing (WES) efforts expanded to analyze over a hundred primary tumors ^20, 21^. A major leap came in 2015 with publication of the data from the PCa branch of The Cancer Genome Atlas (TCGA), a landmark study that published molecular characterization (genomic, epigenomic, and proteomic) of 333 primary prostate tumors ^22^. Another large-scale study (published in 2017) is the Genomic Hallmarks of Prostate Cancer, which includes WGS for 200 primary tumors and WES for an additional 277 ^23^. Two 2018 studies performed WGS on 92 and 93 additional primary tumors, generating more useful data for analysis ^24, 25^. One issue that can arise when comparing datasets from different studies is a lack of uniform pipeline analysis (that is, data standardization, normalization, and statistical cutoffs). A 2018 report sought to tackle this problem by re-analyzing 1,013 available WES datasets (680 primary and 333 metastatic) using a common analysis pipeline ^26^. As a direct result of these transformative studies, researchers finally have an encompassing view of the genetic landscape of primary PCa that provides an important point of reference for understanding this complex disease.

At a broad glance, prostate tumors have, on average, fewer mutations (0.7 per Mb) than other common cancers, such as breast (1.2 per Mb), bladder (7.1 per Mb), colorectal (3.1 per Mb), and melanoma (12.1 per Mb) ^31^. Despite having relatively few point mutations, PCa is characterized by a high rate of genomic instability and chromosomal rearrangments ^32^. The most frequent genomic aberration in primary tumors is a chromosomal rearrangement fusing strong AR-regulated promoters with ETS family genes (62%), resulting in their prominent overexpression ( Table 1) ^22^. Although multiple ETS fusions have been identified, the most common is *TMPRSS2-ERG*, which arises from an approximately 3 Mb deletion on chromosome 21 that brings the androgen-regulated *TMPRSS2* promoter upstream of *ERG* ^22, 33^. In addition, about 3% of primary tumors show mutation/deletion of *ERF*, an ETS repressive cofactor, providing another mechanism for increasing ETS activity without their overexpression ^36, 37^. Other common genomic alterations in primary tumors include loss (usually by deletion) of *PTEN* (17%), point mutations in *SPOP* (11%), mutation or deletion of *TP53* (8%), and amplification of *MYC* (7%) ( Table 1) ^22^. Many of the findings from these sequencing efforts confirmed previously known alterations (for example, *ERG*, *MYC*, and *PTEN*) ^38^, but they also revealed some less-frequent, novel PCa mutations (for example, *SPOP*, *IDH1*, *MED12*, and *FOXA1*) ^21, 22^. The majority of these studies also included mRNA expression or DNA methylation data or both. Information concerning changes in gene expression, mutations, deletions, and amplifications in human PCa can be readily queried via the cBioPortal web tool ( www.cbioportal.org) ^34, 35^.

**Table 1.** Common genomic aberrations in primary prostate cancer.

Gene	Primary tumors altered, percentage	Type of mutation
ETS family ^a^	62	Fusion/Amp
*ERG*	46	Fusion
*PTEN*	17	Homdel/Mut
*SPOP*	11	Mut
*TP53*	8	Homdel/Mut
*MYC*	7	Amp
*AR*	1	Amp
*RB1*	1	Homdel/Mut

A selection of common alterations in primary prostate tumors. cBioPortal ^34, 35^ was used to query the TCGA (The Cancer Genome Atlas) data set, which contains 333 primary tumor samples ^22^. Data were queried specifically for the type of alterations listed in the third column. Amp, genomic amplification; Homdel, homozygous deletion; Mut, nonsynonymous mutation. ^a^ *ERG*, *ETV1/4/5*, *FLI1*.

## Genomic analysis of metastatic tumors

Understanding the genomic landscape of primary tumors has many benefits (for example, understanding tumor origin, aiding prognosis, and revealing therapy options), but there is also a more practical need to understand the lethal form of disease, mCRPC. Androgen deprivation and AR signaling inhibitors (ARSis) (for example, abiraterone and enzalutamide) are initially quite effective, but tumors eventually develop resistance via various mechanisms, including (but not limited to) intra-tumoral androgen synthesis, AR amplification, AR ligand-binding domain mutations, or expression of constitutively active AR splice variants ^4, 39^. Sequencing efforts with metastatic tumors ( Figure 1) identified enrichment of some mutations seen in primary disease, including amplification/mutation of *AR* (61%), amplification of *MYC* (20%), and deletion/mutation of *TP53* (47%) and *PTEN* (41%) ( Table 2) ^27– 30^. mCRPC tumors have about five times as many mutations as primary tumors (2.3~4.4 versus 0.7~1.0 per Mb) ^24, 28, 31^ and include several new mutations, a selection of which is summarized in Table 2. One key advantage of having sequencing data from hundreds of tumors is the ability to use bioinformatics to recognize and cluster low-frequency, recurrent mutations across multiple genes in a single pathway. At the pathway level, mCRPC tumors have frequent alterations in AR signaling (71%), PI3K/PTEN (49%), WNT (18%), cell cycle (21%), and DNA repair (13%) ^28^. Furthermore, about 21% of mCRPC tumors have amplified *HEY1*, which is an important target of the NOTCH pathway ( Table 2) ^38^.

**Table 2.** Common genomic aberrations in metastatic prostate cancer.

Gene	mCRPC altered, percentage	Chromosome	Type of mutation
*AR*	61	Xq	Amp/Mut
ETS family ^a^	49	-	Fusion/Amp
*ERG*	35	21q	Fusion
*TP53*	47	17p	Homdel/Mut
*PTEN*	43	10q	Homdel/Mut
*HEY1*	21	8q	Amp
*E2F5*	21	8q	Amp
*MYC*	20	8q	Amp
*RB1*	17	13q	Homdel/Mut
*FOXA1*	14	14q	Amp/Mut
*CHD1*	11	5q	Homdel/Mut
*FOXO1*	11	13q	Homdel/Mut
*BRCA2*	11	13q	Homdel/Mut
*MED12*	9	Xq	Amp/Mut
*SPOP*	8	17q	Mut
*ATM*	8	11q	Homdel/Mut
*PIK3CA*	8	3q	Amp/Mut
*CDK12*	5	17q	Mut

A selection of common alterations in metastatic castration-resistant prostate cancer (mCRPC). cBioPortal ^34, 35^ was used to query three mCRPC data sets containing 347 tumors from 263 patients ^27– 29^. The second column shows the percentage of patients with a tumor carrying the alteration. Data were queried specifically for the type of alterations listed in the third column. Amp, genomic amplification; Homdel, homozygous deletion; Mut, nonsynonymous mutation. ^a^ *ERG*, *ETV1/4/5*, *FLI1*.

Other recent advances in analyzing mCRPC include circulating tumor cell (CTC) isolation and single-cell sequencing ^40– 42^. In a 2015 report, the authors used single-cell RNA sequencing on 76 CTCs from 12 patients with mCRPC and found enrichment in expression of stem cell genes, non-canonical WNT signaling, and a range of *AR* splice variants, sometimes even within the same cell ^41^. In patients with multiple metastases, tumors usually share common driver mutations and appear to either be clonal or show convergent selection for therapy resistance ^29, 43^. Moreover, one report analyzed sequencing of multiple metastases within patients and observed that many seeded from an earlier metastasis ^43^. The authors also found that metastases within a patient are likely to share tumor suppressor loss-of-function mutations (for example, *PTEN* and *TP53*) but often show unique AR pathway alterations ^43^. Although targeting AR by androgen deprivation and ARSi leads to temporary success, there is a clear need to consider other targets, and these recent genomic studies have helped provide some candidates.

## Three-dimensional culture models

PCa research has advanced with a relatively small collection of commonly used cell lines (and their derivatives), the vast majority of which were isolated from metastatic tumors (for example, LNCaP, VCaP, PC3, and DU145) ^44^. The overwhelming majority of cells in human PCas most resemble luminal epithelial cells and have some basal marker expression ^38^. Unlike the mouse prostate, normal human luminal epithelial cells rarely proliferate and most come from bipotent progenitors in the basal layer ^45^. It is difficult to establish and maintain human luminal epithelial cells in culture; however, luminal-like cells can be differentiated from basal/intermediate cells, which can be maintained in culture ^46– 49^. Extracellular matrix conditions can have a significant impact on cell survival and growth. For example, plating PCa cells on laminin can activate integrin α6 (also known as CD49f), which aids invasion and survival ^50, 51^. It is not clear why primary tumors and normal luminal cells take so poorly to tissue culture conditions. It is likely that something within the *in vivo* microenvironment has not been properly replicated in culture (paracrine factors, cell–cell interactions, and so on). As a way to better mimic the *in vivo* microenvironment, research has expanded into three-dimensional (3D) culture systems.

Prostate cell culture in 3D (that is, spheroids, prostaspheres, and organoids) has aided research by providing more physiologically relevant conditions and allowing more complex cultures ^52– 54^. Organoids can be derived from tumors or normal prostate cells, which can recapitulate basal, intermediate, and luminal cells as well as tumor initiation events such as prostatic intra-epithelial neoplasia (PIN) ^55– 59^. 3D culture can also incorporate different cell types, such as combining epithelial cells plus stroma or cancer cells plus osteoblasts ^60– 62^. Growing primary human tumors in 3D remains a difficult task, but metastatic tumors have been cultured with some success ^52, 58, 63^. Innovative studies using organoid cultures have also improved our understanding of prostate tumor initiation and cell of origin ^64, 65^. A 2016 report provides a protocol for growing prostate organoids using a fairly complex serum-free medium with a variety of growth factors and inhibitors ^52, 58, 66^. Interestingly, many cell lines behave differently in 2D versus 3D culture; for example, LNCaP cells have higher docetaxel resistance in 3D ^67, 68^. Though technically challenging, these new culture methods allow better modeling of normal and tumor epithelial structure. However, better understanding of prostate cell biology is needed so we can more efficiently culture prostate tissues, especially primary tumors.

## Genetically modified mouse models

**Figure 2.**
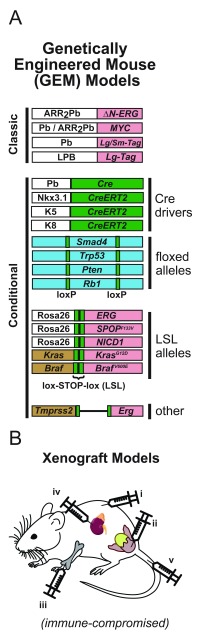
Overview of mouse models of prostate cancer. ( **A**) Genetically engineered mouse models. Classic models use the prostate-specific Probasin (Pb) or ARR _2_Pb promoter to drive expression of oncogenes, including *MYC* ^70, 71^ and N-terminally truncated *ERG* ^71^. In the classic TRAMP model, Pb is used to drive expression of large and small SV40 T-antigen (Tag) ^72^. The LADY models use the Large Pb (LPB) promoter to drive large T-antigen only ^73^. Conditional models use prostate-specific Cre recombinase expression with loxP tagged alleles. Cre is most frequently driven by Probasin (Pb-Cre4 line) or a knock-in tamoxifen-inducible Cre at the *Nkx3.1* locus (Nkx3-1 ^CreERT2^ line) ^74^. Basal (K5) or luminal (K8) keratin promoters can been used to drive layer-specific expression in the prostate but also are expressed in other epithelial tissues ^75, 76^. Flanked loxP (floxed) sites can be used to induce loss-of-function deletions in endogenous tumor suppressor genes, including *Smad4* ^77^, *Trp53* ^78^, *Pten* ^79^, and *Rb1* ^80^. Lox-STOP-lox (LSL) alleles use Cre to remove an upstream STOP codon and allow expression of an oncogene. For constitutive expression, genes can be knocked-in at the ubiquitously expressed *Rosa26* locus (for example, *ERG* ^81^, *SPOP* ^[ *F133V*]^ ^82^, Notch1 intra-cellular domain [NICD1] ^83^). Alternately, mutant genes can be knocked-in at the endogenous locus to maintain normal transcriptional regulation (for example, *Kras* ^[ *G12D*]^ ^84^ and *Braf* ^[ *V600E*]^ ^85^). There is also a model where loxP sites are used to delete the intergenic space between *Tmprss2* and *Erg*, thereby mimicking the fusion observed in tumors ^86, 87^. Color coding: white = promoter, green = Cre or lox, blue = endogenous tumor suppressor, red = oncogene, brown = other endogenous gene. ( **B**) Xenograft models. Cells can be injected into immunocompromised mice via multiple methods: (i) subcutaneous, (ii) prostate (orthotopic for primary tumors), (iii) intra-tibial (orthotopic for bone metastatic tumors), (iv) renal capsule, and (v) tail vein.

Mouse models have been extremely useful for studying disease initiation and progression *in vivo* and can broadly be separated into two categories: genetically engineered mouse (GEM) models and xenograft models ( Figure 2) ^69^. GEM models rely on engineering the mouse genome to knockout or express specific genes, which can be done globally (classic) or in specific tissues (conditional) via tissue-specific promoter-driven expression of Cre recombinase paired with floxed (flanked-loxP) target alleles ( Figure 2A). For prostate-specific expression, the most commonly used promoter is the rat Probasin promoter (Pb), the Large Pb (LPB), or the related ARR _2_Pb, which contains Pb plus enhancer elements for higher expression ^72, 88^. Another common driver for conditional models is a tamoxifen-inducible knock-in Cre (CreERT2) at the *Nkx3.1* locus, which is more specific for prostate luminal cells but carries the caveat of losing one functional copy of the gene ^74^. With CreERT2 models, Cre is still made only in the promoter-specified tissues but must be activated by the addition of tamoxifen (which causes nuclear localization) and thus grants far greater temporal control of recombination. Other valuable CreERT2 drivers include the basal keratins 5 and 14 (K5 and K14) and the luminal keratin 8 (K8) ^75, 76, 89, 90^. These keratin promoters provide basal/luminal specificity in the prostate but are also expressed in many other epithelial tissues. These models have been especially useful for lineage-tracing experiments, which use a brief pulse of tamoxifen to tag epithelial cells with fluorescent proteins and follow them as they divide and differentiate over time ^76^.

There are GEM models matching many of the common alterations observed in human prostate tumors, including *MYC* overexpression ^70, 91^, *Pten* loss ^79, 92^, *ERG* overexpression ^81, 86, 93^, and *SPOP* mutation ^82^ ( Figure 2A). The first-generation mouse models of PCa (TRAMP and LADY) used Pb-driven SV40 T-antigen (Tag), which promotes massive proliferation and creates tumors displaying partial neuroendocrine differentiation ^72, 73^. These models have regained some popularity recently, as neuroendocrine tumors are becoming more common in human PCa. Some prostate GEM models give rise to metastasis, but only one, the LPB-Tag/Pb-Hepsin model expressing SV40 and cell-surface protease Hepsin, reliably metastasizes to bone (up to 40% by 23 weeks of age), the most frequent site in human patients ^77, 94– 96^. Other GEM models used for PCa are included in Figure 2A and have been previously reviewed ^97– 99^. In summary, recent advancements in GEM models enable the study of autochthonously developing PCa in immune-competent animals, but they lack the complexity of human genetics and human prostate biology and rely on contrived genetic manipulations. To study human tumors *in vivo*, experiments rely on xenograft models.

## Xenograft mouse models

With xenograft models, human samples (tissue or cell line) are implanted into immune-compromised mice. Samples can be engrafted via a variety of routes, and the most common is subcutaneous, orthotopic, renal capsule, or tail vein ( Figure 2B) ^100^. Subcutaneous grafts allow easy injection and monitoring of tumor growth, while orthotopic injections benefit from a proper microenvironment at the cost of more difficult injection and monitoring. Orthotopic injections can be made into the prostate (for primary tumors) or the metastatic site, including intra-tibial injections for studying bone metastasis ( Figure 2B) ^101^. Renal capsule implant is somewhat of a compromise between subcutaneous and orthotopic: a moderately difficult grafting site that is favorable for prostate tissue growth ^100^. Lastly, tail vein injections require single-cell suspensions and enable investigation of extravasation and metastasis establishment ^102^.

Patient-derived xenografts (PDXs) specifically use human tumor samples for engrafting into mice ^63, 103^. These models allow propagation of tumors (metastatic and primary) that do not grow well in culture; however, PDX tumors require continual passage in mice, which adds considerable cost ^63^. The PCa field has had successful PDX models since the late 1990s, but the procedure is laborious and most reports describe only few (<10) established lines ^104^. The number of available PDX lines was greatly expanded with the LuCaP series, which was first reported in 1996 with two lines and currently consists of 21 ongoing founder lines from a variety of samples, including four primary tumors and 17 metastases ^63, 105^. Moreover, 10 of the lines have undergone castration in mice to yield castration-resistant variants ^63^. The overall initial take rate of the LuCaP PDX lines was about 10% and, once established, most lines have a take rate of about 60% to 80% and take 4 to 16 weeks to reach maximum size (~1,000 mg). Genomic analysis of the LuCaP tumors revealed that most maintained their genomic profile from the original patient sample ^63^. The lines contain a variety of hallmark mutations, including *AR* amplification (eight lines), *PTEN* loss (eight heterozygous and four homozygous), *RB1* loss (10 heterozygous and six homozygous), *TMPRSS2-ERG* fusion (10), *BRCA2* homozygous loss (one), and neuroendocrine subtype (four) ^63^. PDX models continue to evolve and enable researchers to test a variety of therapeutic strategies against a range of genomic tumor backgrounds and to better understand tumor resistance mechanisms.

## Progress in understanding the role of ETS factors

**Figure 3.**
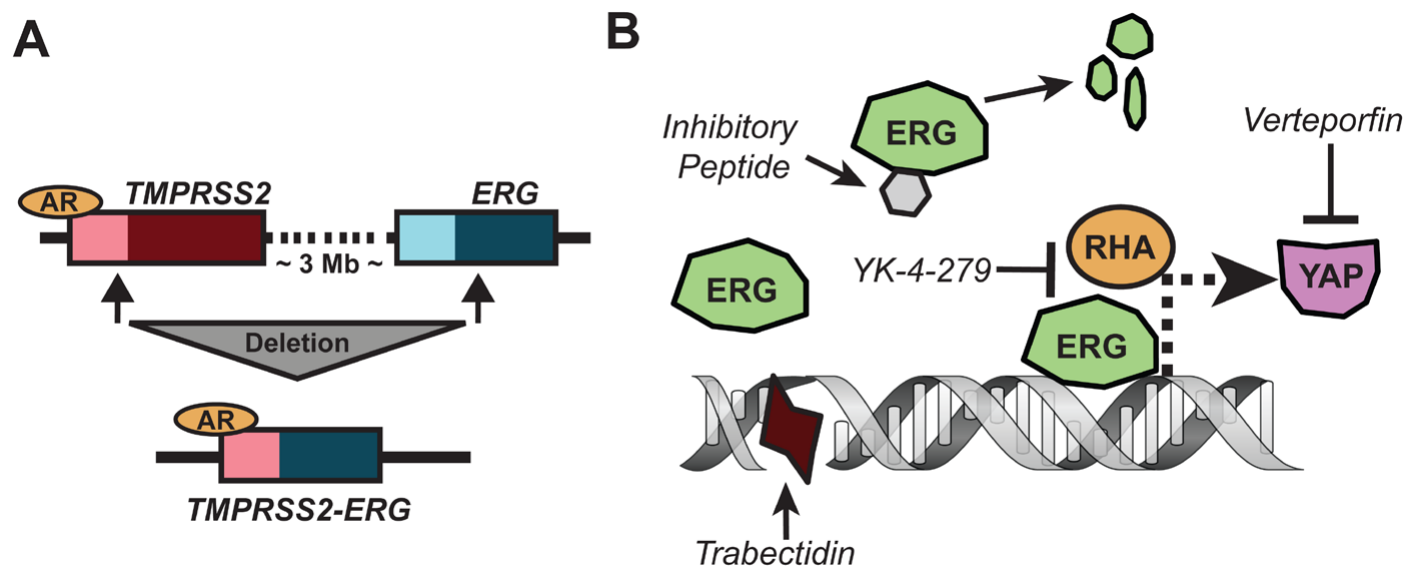
Overview of ERG fusion and targeted therapies. ( **A**) The most common ETS fusion arises from a 3 Mb deletion on chromosome 21, which brings the androgen receptor (AR)-regulated *TMPRSS2* promoter (light red) upstream of the *ERG* gene (dark blue), usually clipping the first three to five exons of *ERG* in the process. ( **B**) A schematic showing mechanisms of anti-ERG therapies. Inhibitory peptides block ERG binding to DNA and cause protein destabilization ^110^. Verteporfin blocks YAP1, a downstream target of ERG ^93^. YK-4-279 blocks ERG interaction with RNA helicase A (RHA), thereby disrupting transcription of targets ^111, 112^. Lastly, trabectedin binds minor grooves in ERG binding sites and disrupts its binding to target promoters ^113^.

One of the unique genomic alterations in PCa is the recurrent fusions involving strong AR-regulated promotors to ETS family transcription factors (most frequently, *ERG*) ^27, 33^. In normal prostate tissue, *ERG* is expressed at very low levels, but it is overexpressed in PIN and adenocarcinoma ^106, 107^. The most frequent fusion (caused by a 3 Mb deletion) joins the *TMPRSS2* promoter upstream of *ERG* ( Figure 3A), although other fusions have been observed with alternate promoters (for example, *FOXA1*, *FOXP1*, *EST14*, and *HERVK17*) and ETS family members ( *ETV1*, *ETV4*, *ETV5*, and *FLI1*) ^22, 27, 108^. Likewise, loss of the ETS family transcriptional repressor *ERF* (though much less common) can also stimulate oncogenic ETS activity ^36^. ETS fusions are observed at similar frequencies in primary and mCRPC tumors ^28^, tend to co-occur with *PTEN* loss ^22^, and are mutually exclusive with each other and *SPOP* mutations ^21, 22, 82^. Though ETS factors (especially *ERG*) are frequently altered in PCa, research is ongoing to understand exactly what role they play in disease initiation or progression or both.

GEM models have been used as one way to investigate the role of *ERG* in PCa development. A 2007 report used mice with Pb-driven overexpression of *ETV1* (Pb- *ETV1*) and observed PIN ^109^. Other researchers found that high *ERG* overexpression leads to PIN lesions and disorganization of the basal cell layer in adult animals, that more lesions form as the mice age, and that there is a partially penetrant tumor phenotype in old (16- to 24-month-old) mice ^71, 93^. The expression level of *ERG* is critical, as mouse strains with lower levels of *ERG* overexpression do not develop tumors ^71^. One study found that expression of *ERG* in heterozygous *Pten* mice causes high-grade PIN at 2 months and invasive carcinoma by 6 months and that heterozygous *Pten* mice without *ERG* developed PIN at 8 months and no adenocarcinoma ^114^. Similarly, compared with *Pten* control mice, mice with conditional prostate-specific deletion of *Pten* and overexpression of *ERG* display statistically significant acceleration of prostate tumor development ^81^. Thus, *ERG* overexpression is a weak driver of tumor development in mice, but it can accelerate tumor development in the context of *Pten* downregulation ^114, 115^.

Another avenue of research involves defining specific targets and mechanisms of ETS factors in prostate tumorigenesis. There is evidence that *ERG* can act in part by modulating AR transcriptional activity, although the mechanism may depend on *PTEN* status ^81, 116, 117^. *ERG* has also been reported to positively regulate *MYC* ^115, 118^ and *NOTCH* ^119^, both of which are key for prostate differentiation and tumor development, suggesting that *ERG* may have a role in disrupting terminal differentiation ^38, 120, 121^. In addition, *ERG* overexpression leads to increased endoplasmic reticulum stress in LNCaP cells and the prostates of aged Pb- *ERG* mice ^122^. Transcriptional analysis of *ERG* overexpression in mice revealed upregulation of a *YAP1* gene signature, suggesting an interaction with the Hippo tumor suppressor pathway ^93^. Mechanistically, it was found that *YAP1* and *TAZ*, the transcriptional effectors negatively regulated by the Hippo pathway, are normally expressed at very low levels in human prostate luminal cells but they can be transcriptionally re-activated by overexpressed *ERG* and *ETV1* ^93, 123– 125^. Knockdown and constitutive activation experiments established *YAP1* as a key functional target of *ERG* in prostate cells *in vitro* ^93, 123– 125^. Moreover, expression of constitutively active YAP1 in the mouse prostate is sufficient to drive PIN and partially penetrant tumor formation in older mice, similar to the effects of *ERG* overexpression ^93^. Knockdown of *Erf* in mouse prostate organoids upregulates an ERG signature and, when combined with *Pten* knockout, leads to tumor formation upon subcutaneous engraftment ^36^. With these recent studies, some of the functions of *ERG* in PCa are becoming clearer, although much remains to be discovered. Overall, ETS factors appear to be important drivers in PCa development, but their full effects may be seen only in the context of other alterations (for example, *AR*, *PTEN*, *MYC*, and *NOTCH*), and cross-talk between these pathways is still being investigated.

## Targeting of ETS factors

Direct targeting of transcription factors is notoriously difficult but not impossible ^126^. One route is to target a downstream effector of ERG that is more amenable to inhibitors, such as YAP, which can be inhibited by verteporfin ( Figure 3B). Verteporfin treatment decreases VCaP (ERG ^+^) xenograft tumor growth in mice ^93^. In efforts to target ERG directly, phage-display library screens have been used to identify 12 ERG inhibitory peptides ^110^. Two of the peptides were modified (to improve cell permeability and localize to the nucleus) and tested *in vitro* and *in vivo*. The peptides disrupted DNA binding, destabilized ERG ( Figure 3B), decreased VCaP and PC3-ERG invasion, and reduced VCaP xenograft growth ^110^.

Important lessons may be learned from another cancer type with recurrent ETS fusions, Ewing’s sarcoma, in which more than 90% of tumors are driven by a rearrangement fusing the *EWSR1* gene to *FLI1*, a paralog of *ERG* ^127, 128^. ETS fusions do occur in some other cancers, but it is not a frequent event ^129^. YK-4-279, a FLI1 inhibitor initially developed for Ewing’s sarcoma, was reported to shrink PCa tumors in ERG ^+^ mouse xenograft models ( Figure 3B) ^111, 112^. Another potential candidate from the Ewing’s sarcoma field is trabectedin (and its second-generation analogue lurbinectedin), which works in part by binding DNA minor grooves in ETS binding sites and disrupting EWS-FLI1 binding at target promoters ( Figure 3B) ^113, 130, 131^. Very few studies (and only two phase II clinical trials) have investigated trabectedin in PCa, but the results were disappointing ^132, 133^. However, patients were not initially stratified by ERG status and the study did not use the newer drug lurbinectedin. These studies demonstrate that there are multiple ways to target ERG, directly or indirectly, and these therapies may be an effective option for patients with ETS ^+^ prostate tumors.

## DNA repair mutations in prostate cancer

**Figure 4.**
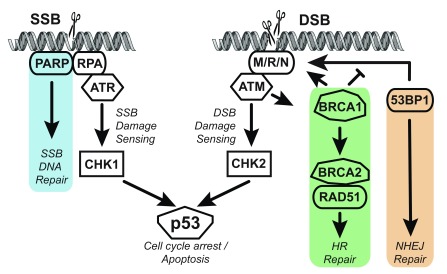
Simplified DNA repair pathway diagram. Single-strand breaks (SSBs) are recognized by a handful of proteins, including poly (ADP-ribose) polymerase (PARP) and RPA. PARP helps recruit DNA repair machinery to repair SSBs (blue shading). ATR is recruited to the site of damage and activates (phosphorylates) a variety of damage-sensing mediators, including CHK1, which in turn can activate p53 and, depending on other signals, pause the cell cycle until damage is repaired or induce apoptosis. Double-strand breaks (DSBs) recruit a variety of factors, including the M/R/N complex (MRE11, RAD50, and NBS1). This complex recruits and activates ATM, which phosphorylates DSB-sensing mediators, including CHK2. 53BP1 binds to M/R/N on loose DNA ends and promotes non-homologous end joining (NHEJ) repair (orange shading). During late S/G _2_ phase, BRCA1 can be activated by ATM and compete with 53BP1 for binding at the M/R/N complex and aid resection of DNA ends to promote homologous recombination (HR) repair (green shading).

Despite having a low burden of point mutations compared with other cancers, PCa has a high rate of genomic instability (amplifications, deletions, and chromosomal rearrangements) ^32^. Genomic instability is a result of DNA damage, which can arise from many sources, including (but certainly not limited to) DNA replication stress, alkylating agents, mitotic chromosome segregation errors, and radiation ^23, 134, 135^. DNA can also be damaged as a result of transcriptional stress; AR has been reported to recruit topoisomerase enzymes to counter DNA torsional stress caused by transcription and enhancer looping ^136, 137^. An extreme form of genomic instability is chromothripsis, which occurs in about 20% to 30% of primary prostate tumors and involves acute chromosome shattering and reassembly, causing deletions and rearrangements ^23, 138^. Damage that breaks the phosphate backbone or requires repair via base excision repair, mismatch repair, or nucleotide excision repair will lead to single-strand breaks (SSBs) ^134^. If SSBs occur close together on opposite strands, double-strand breaks (DSBs) can occur, which are more severe and must be repaired by homologous recombination (HR) or non-homologous end joining (NHEJ) ( Figure 4). HR can occur only if a sister chromatid is present (late S or G _2_ phase) and uses the non-damaged DNA as a template for error-free repair of the damaged chromatid, whereas NHEJ can repair DSBs at any cell cycle stage (predominantly G _0_/G _1_) but has the possibility of introducing deletions or insertions ^139, 140^. Further information on DNA damage-sensing and repair mechanisms can be found in several recent reviews ^134, 141, 142^.

Germline mutations in DNA repair genes are responsible for a variety of human hereditary diseases, many of which include a predisposition to cancer ^143, 144^. Two such genes are the key signaling kinases in the DNA damage response—ATM (primarily activated by DSB) and ATR (primarily activated by SSB)—which can activate other signaling proteins, including CHK1 ( *CHEK1*) and CHK2 ( *CHEK2*) ( Figure 4) ^142^. Two other important DNA repair genes are *BRCA1* and *BRCA2*, for which single-copy germline mutations increase the risk of multiple cancers, most significantly breast and ovarian cancer ^145– 147^. Mechanistically, BRCA1/BRCA2 are crucial for recruiting RAD51, which is required for HR. BRCA1 has multiple roles, including promoting loose-end resection and aiding RAD51 loading onto DNA ^148^. A recent study suggests that BRCA1 competes with 53BP1 for binding at DSBs and helps determine whether repair is shunted toward NHEJ or HR ( Figure 4) ^149^.

In 2016, a multi-institutional study sequenced the germline DNA of nearly 700 men with mCRPC and observed that 11.8% of patients carried a germline mutation in a DNA repair gene, most frequently *BRCA2* (5.3%), *CHEK2* (1.9%), or *ATM* (1.6%) ( Table 3) ^150^. Furthermore, somatic metastatic tumor sequencing through the SU2C/PCF landscape project determined that about 20% of metastatic tumors have a DNA repair gene aberration ^28^. *BRCA2* germline mutations occur at about 0.3% in the general population and, though not enriched among all primary tumors, correlate with high-grade disease ^22, 150– 152^. For example, *BRCA2* tumors are more likely to show a pattern of intra-ductal carcinoma, which involves large tumor-filled prostate ducts with intact basal layers and correlates with poor prognosis ^153– 155^. This knowledge has led to an ongoing discussion about whether all men who present with metastatic PCa should be screened for BRCA status as well as those with localized disease where biopsies demonstrate intra-ductal carcinoma patterning ^154– 156^.

**Table 3.** DNA repair mutation rate in tumors and germline.

Gene	Tumor		Germline		
	Primary	mCRPC	Overall	Primary	mCRPC
*ATM*	**6**	**8**	**0.3**	**1**	**1.6**
*ATR*	**0.3**	**0.8**	**0.1**	**0**	**0.3**
*CHEK2*	**3**	**4**	**0.6**	**0.4**	**1.9**
*BRCA1*	**1.2**	**0.8**	**0.2**	**0.6**	**0.9**
*BRCA2*	**3**	**11**	**0.3**	**0.2**	**5.4**

Mutation frequencies (percentages) for selected DNA repair genes in primary tumors and metastatic castration-resistant prostate cancer (mCRPC) and in the germline of normal, primary, or mCRPC patients. Tumor mutation frequencies were calculated the same way as in Table 1 and Table 2. Germline mutation frequencies include 692 mCRPC patients, 499 primary, and 53,105 overall (exome aggregation consortium) ^150^. Shading: light blue: 0–0.9%, dark blue: 1–2.9%, light red: 3–4.9%, dark red: ≥5%.

Other studies have confirmed similar rates (about 8–12%) of germline DNA repair defects in patients with mCRPC but had conflicting results as to whether germline mutant patients respond better to anti-androgen therapy ^157– 160^. Providing evidence against better outcomes are reports that germline mutant patients did not respond any better to initial androgen deprivation ^157^ and patients with mCRPC saw no additional benefit from abiraterone or enzalutamide ^158^. However, other studies observed that DNA repair-deficient mCRPC tumors responded better to abiraterone ^160^ and mCRPC patients with germline *BRCA1/BRCA2/ATM* mutations showed a better rate of greater than 90% prostate-specific antigen (PSA) reduction (78% versus 28%) and overall survival at 4 years (~75% versus ~25%) on ARSi therapy ^159^. Although germline DNA repair mutations account for only about 12% of patients with mCRPC, it will be important to better understand how these patients will respond to anti-androgen therapies. Furthermore, the identification of DNA repair mutations (whether germline or somatic) may open a window to new therapeutic options for thousands of the roughly 30,000 men who succumb to metastatic disease every year in the US and also identify family members at increased risk for cancer ^1^.

## Targeting poly(ADP-ribose) polymerase

For patients with tumors deficient in DSB repair, there is strong rationale for targeting poly(ADP-ribose) polymerase (PARP), a family of proteins that are required for sensing and repairing SSBs ( Figure 4) ^161^. Without PARP, SSBs will cause stalling of replication forks during DNA replication that leads to DSBs, which then require HR or NHEJ for repair. Thus, cells lacking *BRCA1* or *BRCA2* must rely on error-prone NHEJ for DSB repair and are highly sensitive to loss of PARP ^161– 163^. In 2014, the US Food and Drug Administration (FDA) approved olaparib, a PARP inhibitor (PARPi), for the treatment of BRCA-mutant ovarian cancer, where it was found to extend average progression-free survival from 4.3 to 11.2 months ^164^. Since then, two other PARPis have received FDA approval for BRCA-deficient ovarian cancer: rucaparib and niraparib. Currently, those and other PARPis are in various clinical trials and studies are investigating whether the combination of PARPis with DNA-crosslinking drugs (that is, platinum-based chemotherapeutics) will yield better patient outcomes ^161, 165^. Testing of PARPis in PCa patients with BRCA mutations is ongoing. The TOPARP trial (Trial of PARP Inhibition in Prostate Cancer) tested olaparib in men with mCRPC and reported exciting preliminary results demonstrating response rates of 88% (14/16) in those men with a DNA repair defect and 6% (2/33) in those men without, strongly supporting treatment stratification based on DNA repair deficiency ^166^. Currently, searches for “PARP inhibitor” in “prostate cancer” on ClinicalTrials.gov yield 13 clinical trials; only two of these trials are completed, and there are no public results yet ^167, 168^.

Although most studies are ongoing, a 2018 report used retrospective analysis of an earlier study ^150^ to examine whether PCa patients with germline DNA repair defects had different responses to ARSi, docetaxel, or PARPi ^158^. Of the previously treated patients, 60 out of 390 had germline mutations and 36% of those were treated with PARPi (some with platinum chemotherapeutic as well). Germline status had no statistically significant correlations with response to docetaxel, ARSi (abiraterone/enzalutamide), or PARPi ^158^. Being a retrospective analysis, this study comes with multiple caveats, including the lack of initial patient stratification, inconsistent treatment methods, and a relatively small number of patients with germline mutations who received PARPi treatment (total of 22, of which 16 were *BRCA2* mutant). In addition, the study did not have information about the tumor mutation landscape, so there could yet be a correlation between DNA repair mutations and PARPi response that was masked by confounding factors (for example, ETS fusion, *AR*, or *PTEN* status) ^158^. Ongoing trials may yet prove to be beneficial for patients with germline DNA repair defects, and further research is needed to better understand how these mutations affect response to androgen deprivation and other PCa therapy resistance.

## SPOP mutation

**Figure 5.**
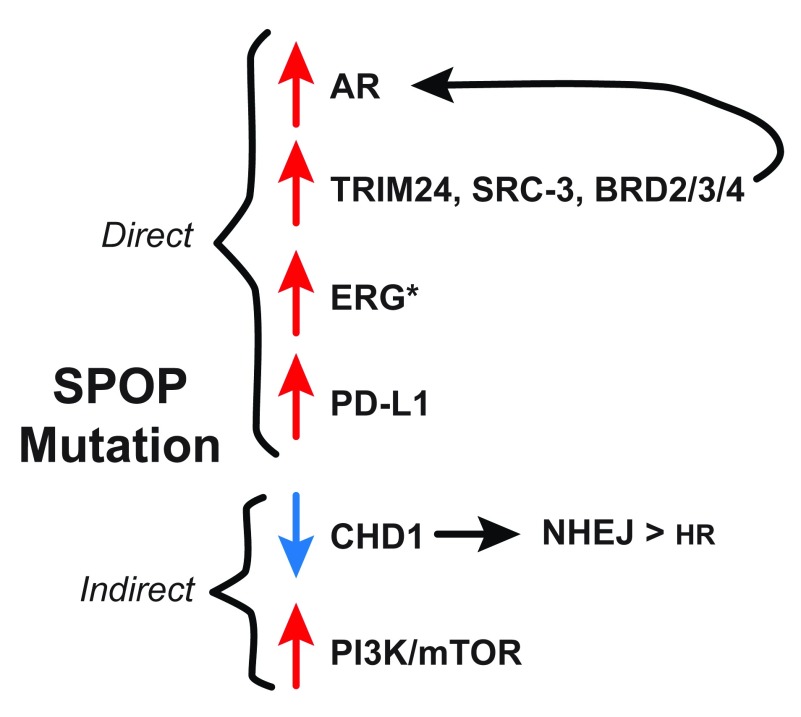
SPOP mutations and prostate cancer. SPOP adapts the CUL3 E3-ligase complex to directly target and degrade AR and the AR co-activators TRIM24, SRC-3, and BRD2/3/4 ^170– 176^. Thus, loss of SPOP increases both AR and its cofactors, leading to increased AR signaling. (*) ERG has also been reported as a direct SPOP target, although this is a debated topic ^176– 178^. SPOP has been shown to target PD-L1, a key target of checkpoint inhibitor immunotherapies ^179^. For indirect mechanisms, *SPOP* mutation strongly correlates with deletion of *CHD1*, which leads to 53BP1 stabilization and preference for error-prone NHEJ DSB repair ^58, 180^. *SPOP* mutation also upregulates PI3K/mTOR signaling via an unknown mechanism, which aids tumor growth and survival ^83^. AR, androgen receptor; DSB, double-strand break; NHEJ, non-homologous end joining.

One of the novel PCa alterations elucidated by genomic sequencing efforts is mutation of *SPOP* ^21^. Heterozygous point mutations in *SPOP* occur in about 10% of primary and metastatic tumors ( Table 1 and Table 2) and are mutually exclusive to *PTEN* loss and ETS rearrangements. SPOP is an adapter component of the CUL3 E3-ligase complex, which has multiple degradation targets, including AR and its co-activators SRC-3 ( *NCOA3*) and TRIM24 ( Figure 5) ^169– 172^. Other recently identified direct targets of SPOP are the BET family proteins: BRD2/3/4 ^173– 175^. BET proteins are transcriptional co-activators that upregulate a variety of oncogenes, including *AR*, *MYC*, and *ERG* ^173^. BET inhibitors are being investigated for use in PCa ^181^, but *SPOP* mutant tumors with elevated BET expression may be more resistant to such therapies ^173, 181^. Earlier this year, PD-L1 was also identified as an SPOP target, which has implications for immunotherapy and will be discussed later ^179^.

ERG has also been reported as an SPOP target, although there are conflicting data in the literature for this relationship ^176– 178^. In support of the connection, two reports from 2015 identified ERG as a direct SPOP degradation target. One report found that knockdown of *SPOP* or *CUL3* in PC3 and DU145 cells increased ERG (but not ETV1 or ERF) protein expression by increasing protein half-life and that this could be blocked by inhibition of casein kinase I ^177^. The authors also used a tissue microarray with immunohistochemistry (IHC) (for ERG expression) and *in situ* hybridization (for *TMPRSS2-ERG* fusions) to identify 14 ERG-positive/fusion-negative primary tumors, of which five (36%) had *SPOP* mutations, suggesting that *SPOP* mutation may upregulate ERG without fusion events ^177^. The second study found that *SPOP* knockdown increased ERG in LNCaP, C4-2, PC3, and 22RV1 lines ^176^. Both studies reported that SPOP targets a recognition site coded by the fourth exon of *ERG* that is usually disrupted in *TMPRSS2-ERG* fusions ( Figure 3), thus suggesting that fusions not only cause transcriptional upregulation but also increase protein stability ^176, 177^.

In 2017, a study using a GEM model of *SPOP* mutation (F133V) observed high-grade PIN at 6 months in *Pten ^−/+^* mice and prostate tumors in *Pten ^−/−^* mice by 12 months ^82^. Additionally, the authors found that *SPOP* mutation alone increases PI3K/mTOR signaling. Furthermore, while PI3K signaling has negative feedback on *AR*, *Pten ^−/−^/SPOP ^F133V^* tumors maintain Ar and express high levels of its transcriptional targets ( *Fkbp5*, *Psca*, and *Nkx3.1*) ^82^. Thus, SPOP appears to have key intersections between AR and PI3K pathways which may help explain its importance in PCa ( Figure 5). These findings, however, do not provide a reasonable explanation for the mutual exclusivity between *PTEN* loss and *SPOP* mutation in human PCa. In a 2018 report from the same group, the authors investigated potential effects on ERG with their GEM model. Unlike the investigators in the 2015 studies ^176, 177^, who observed that *SPOP* mutation or knockdown in PCa lines increased ERG protein, this group did not observe upregulated Erg or an Erg transcriptional signature in their *SPOP ^F133V^* mice ^178^. In addition, they detected ERG (by IHC) in only one out of 22 SPOP-mutant human tumor samples, leading them to conclude that mutant SPOP was not regulating ERG ^178^. One important lesson from these conflicting studies is that loss of SPOP protein does not appear to have the same functional consequence as overexpression of a mutant. Importantly, *SPOP* is rarely deleted in PCa tumors and the vast majority of mutations are in-frame missense point mutations. Clearly, additional research is needed to fully characterize SPOP mutants and understand how they differ in their interactions with wild-type SPOP targets.

*SPOP* has also been linked to DNA repair. *SPOP* mutant tumors have especially high rates of chromosomal rearrangements and share a transcriptional signature with *BRCA1* loss, implicating *SPOP* in genomic instability ^182^. Furthermore, one of the common associations with *SPOP* mutations is deletion of *CHD1*, which is involved in DSB repair and whose loss correlates with poor survival ^24, 180, 183^. A query on cBioPortal using all available PCa datasets shows that *CHD1* loss and *SPOP* mutation are significantly correlated ( *p* <0.01). Specifically, 57% of tumors with *CHD1* deletion (68/119) have an *SPOP* mutation and 29% of tumors with *SPOP* mutations (68/233) have *CHD1* deletion. A 2017 report demonstrated that *CHD1*-null cells (mouse stem cells and 22RV1) are more sensitive to olaparib (PARPi) and carboplatin and have increased 53BP1 protein stability, which promotes error-prone NHEJ repair ( Figure 4) ^180^. Furthermore, *PTEN* deletion is mutually exclusive to *CHD1* deletion, and PTEN-null tumors require *CHD1* for proliferation and survival ^184^. Thus, *CHD1* loss may partly explain why *SPOP* mutations are exclusive of *PTEN* loss. In summary, *SPOP* has been implicated in many key PCa pathways (AR, MYC, ERG, PI3K, and DNA repair) and work has only recently begun to uncover specific targets and oncogenic mechanisms. Better understanding of *SPOP*, including its connections with *ERG* and *CHD1*, will be needed to help choose successful targeting strategies.

## Androgen receptor-negative prostate cancer

The vast majority of prostate tumors depend on the AR pathway for survival ^28^. However, there are small subsets of PCa whose frequency appears to be increasing, including neuroendocrine PCa (NEPC) ^185^ and double-negative PCa (DNPC) ^186^, which lack AR expression and therefore are not sensitive to androgen deprivation or ARSi.

Primary prostate tumors often show regions of neuroendocrine foci, but predominantly NEPC tumors (also referred to as ‘small cell’) are rare at initial diagnosis (<2%) ^5, 187, 188^. However, since the advent of new ARSi therapies (abiraterone in 2011 and enzalutamide in 2012) ^189, 190^, there has been an increase in NEPC, which now accounts for about 15% of mCRPC and has become a mechanism of ARSi resistance ^185, 186, 191^. These tumors are typically more aggressive and are characterized by their lack of AR and expression of neuroendocrine-associated genes, such as chromogranin A ( *CHGA*) and synaptophysin ( *SYP*) ^5, 185^. NEPC tumors often show upregulation of stem-associated genes (for example, *SOX2* and *MYCN*) ^192– 194^ and upregulation of genes associated with epithelial–mesenchymal transition (for example, *SNAI1* and *VIM*) ^5^ and frequently lose expression of *TP53* and *RB1* ^195^. Moreover, these molecular changes are also directly implicated in resistance to AR-targeting therapies ^192, 195^. Interestingly, NEPC tumors have *TMPRSS2-ERG* fusions at about the same rate as adenocarcinomas; however, owing to the lack of AR signaling, the expression levels of ERG are low in these tumors. These findings suggest that NEPC represents trans-differentiation from an androgen-responsive, epithelial-derived precursor, as opposed to the possibility that they originate from normal neuroendocrine cells, which make up less than 1% of cells in the normal prostate ^38, 185^.

In addition to NEPC, there is a recently identified subtype called DNPC that lacks AR and NEPC markers ^186, 196^. For example, recent RNA sequencing and pathway analysis with mCRPC samples from 96 patients treated before or after 2012 (the advent of abiraterone/enzalutamide) identified an increase in patients with NEPC (6.3% to 13.3%) as well as an even larger increase in DNPC (5.4% to 23.3%) ^186^. It is also possible that DNPC is not an entirely distinct subset of PCa but rather an intermediate step on the way from adenocarcinoma to NEPC. An AR-negative LNCaP line (LNCaP ^APIPC^), which lacks CHGA and SYP, was used to investigate the mechanisms driving DNPC. This line has diminished AKT signaling and relies on an upregulated autocrine FGF8 → FGFR → ERK signaling pathway for survival ^186^. Analysis of human tumor data and PDX lines with DNPC confirmed a pattern of upregulated FGFs (FGF1/8/9), FGFRs (FGFR1/2/3/4), and an ERK signature. Furthermore, LNCaP ^APIPC^ xenografts are sensitive to FGFR inhibitors (CH-5183284, PD173074) ^186^. Thus, targeting the FGFR/ERK signaling axis may be beneficial for patients with DNPC, although it has yet to be tested in clinical trials. Thus, while AR-negative PCa accounts for a minority of prostate tumors, they are becoming more common and will require a different therapeutic strategy than classic AR-positive PCa.

## Immunotherapy

Recently, cancer immunotherapy has received significant attention and has demonstrated great potential across different types of cancer. Immunotherapies can broadly be grouped into three strategies: cancer vaccines, immune checkpoint inhibitors, and engineered live immune cell components. Cancer vaccines use tumor-specific proteins to generate a targeted immune response or tag tumors with a lethal, targetable protein. There are a handful of vaccine-based trials for PCa, although the only currently FDA-approved therapy is Sipuleucel-T (also known as Provenge ^®^) ^197^. Sipuleucel-T uses prostatic acid phosphatase as a tumor antigen and has shown about 4- to 5-month extended survival for patients with mCRPC, while patients with lower baseline PSA level had even greater (about 13-month) survival ^198, 199^. Several additional PCa vaccines are being tested, and detailed information about those studies can be obtained in the cited reviews ^197, 200^.

A second branch of immunotherapy is checkpoint inhibition, which attempts to re-activate cancer-targeting T cells that have been disarmed by tumors. The primary targets for checkpoint inhibition are CTLA-4, PD1, and the PD1 ligands PD-L1/PD-L2. An early checkpoint inhibitor is ipilimumab, which targets CTLA-4 and was approved by the FDA in 2011 for the treatment of melanoma. Ipilimumab has been tested in PCa trials with mixed results; it was able to delay progression but did not extend overall survival ^197, 201^. However, this study had a small subset of patients (two out of 400) who had a complete response (>4 years) ^202^. Other checkpoint inhibitors (for example, nivolumab, pembrolizumab, and atezolizumab) target PD1 or PD-L1/L2 ^200^. In general, tumors with high mutational burden generate more novel proteins (neoantigens) and respond more favorably to immunotherapy ^203, 204^. A small subset of mCRPC tumors have mismatch repair defects (5% rate of deletion/mutation of *MSH2* or *MSH6* or both) ^27– 29^ and exhibit a hypermutated phenotype ^205^. Interestingly, mismatch repair defects appear to be enriched in the most aggressive primary tumors. In one study, 40% of samples with intra-ductal carcinoma (four out of 10) showed loss of *MSH2*, *MSH6*, or *MLH1* ^206^. Another investigation analyzed 1,133 primary and NEPC tumors by IHC via tissue microarray and observed that 8% of Gleason pattern five tumors (seven out of 91) had loss of MSH2 protein (due to technical issues, MSH6 and MHL1 were not included) ^207^. In 2017, the FDA approved pembrolizumab for the treatment of solid metastatic tumors with mismatch repair-defects, providing a new option for some patients with mCRPC ^208^.

A recent study suggests that even tumors with low PD-L1 may be targetable using combination therapy. CDK4 was reported to negatively regulate PD-L1 via SPOP, and CDK4/6 inhibitors can cause upregulation of PD-L1 in mouse tissue and breast cancer xenografts ^179^. The authors went on to show that 80% of SPOP-mutant tumors (12 out of 15) had high PD-L1 by IHC staining versus 10% of the non-mutant SPOP tumors. Thus, SPOP-mutant tumors are likely to express PD-L1 and benefit from checkpoint inhibitory therapy, while other tumors may be driven to express PD-L1 by CDK4/6 inhibition.

Another recent report focused on targeting myeloid-derived suppressor cells (MDSCs), which can shield tumors from T cells ^209^. A GEM model with Pb-driven knockout of *Pten*, *Trp53*, and *Smad4* was developed, and combinations of checkpoint inhibitors (anti-CTLA4 and anti-PD1 antibodies) plus inhibitors against multiple tyrosine kinases (dasatinib and cabozantinib) and PI3K (dactolisib/BEZ235) were evaluated. The authors found that combination therapy (checkpoint + tyrosine kinase + PI3K inhibition) had a major effect on decreasing tumor burden. Furthermore, they went on to discover that a key mechanism of this therapy was decreased cytokine production and MDSC tumor infiltration caused by the inhibition of tyrosine kinases and PI3K, which in turn sensitized the tumors to the checkpoint inhibition ^209^.

The third branch of immunotherapy uses engineered immune cells, including chimeric antigen receptor T-cell (CAR-T) therapy. CAR-T involves isolating patient immune cells and genetically engineering them to express a chimeric protein fusing a tumor-recognizing antibody region with a T-cell activation domain ^210^. The engineered cells are then grafted back into the patient. CAR-T cells can directly recognize tumors and trigger activation. This therapy has been extremely successful for treating B-cell acute lymphoblastic leukemia and B-cell lymphoma ^210, 211^. Trials with CAR-T therapies for PCa are under way and primarily involve using PSMA and PSCA as targeting antigens ^212, 213^. The multiple immunotherapy strategies of cancer vaccines, checkpoint inhibitors, and CAR-T continue to improve. Meanwhile, patient stratification based on tumor mutational burden, PD1/PD-L expression, and tumor-enriched antigens such as PSMA and PSCA will help direct these therapies to the patients most likely to benefit.

## Conclusions

Owing to large-scale sequencing efforts ( Figure 1), the PCa field now has a near-comprehensive view of the mutational landscape of human PCa. These studies revealed that ETS fusions are the most frequent mutation in primary tumors, occurring in a little over half of cases ( Figure 3). Likewise, the second most common alteration, *PTEN* loss, occurs in about a quarter of primary tumors. PCa shows great resilience in evading androgen deprivation therapy and finding ways to maintain the AR pathway, but there is a growing number of tumors that do not express AR and rely on alternate survival mechanisms ^4^.

Perhaps the key takeaway is that human prostate tumors are driven by a combination of alterations in a handful of signaling pathways. Understanding the role of these pathways in tumor initiation, progression, and therapeutic resistance will be critical in the future. In order to functionally test the role and mechanisms of these signaling pathways, there is a need to continually improve existing cell culture and animal models. Recently developed GEM models and organoid culture conditions can provide great opportunities for studying disease initiation, metastasis, and testing therapies against patient-derived tumors. As has become clear with other cancers, there will almost certainly be no single effective therapy for PCa. While most tumors will likely still benefit from improved AR-targeting therapies, it will be important to recognize subsets of tumors that may benefit from targeting other supporting mutations.

## Abbreviations

AR, androgen receptor; ARSi, androgen receptor signaling inhibitor; CAR-T, chimeric antigen receptor T-cell; CTC, circulating tumor cell; DNPC, double-negative prostate cancer; DSB, double-strand break; FDA, US Food and Drug Administration; GEM, genetically engineered mouse; HR, homologous recombination; IHC, immunohistochemistry; LPB, large probasin promoter; LSL, Lox-STOP-Lox; mCRPC, metastatic castration-resistant prostate cancer; MDSC, myeloid-derived suppressor cell; NEPC, neuroendocrine prostate cancer; NHEJ, non-homologous end joining; PARP, poly(ADP-ribose) polymerase; PARPi, poly(ADP-ribose) polymerase inhibitor; Pb, Probasin; PCa, prostate cancer; PDX, patient-derived xenograft; PIN, prostatic intra-epithelial neoplasia; SSB, single-strand break; TCGA, The Cancer Genome Atlas; WES, whole exome sequencing; WGS, whole genome sequencing.
